# Adaptive Transition-State Refinement with Learned Equilibrium Flows

**DOI:** 10.1021/acs.jcim.5c02902

**Published:** 2026-02-02

**Authors:** Samir Darouich, Vinh Tong, Tanja Bien, Johannes Kästner, Mathias Niepert

**Affiliations:** † Institute for Theoretical Chemistry, 9149 University of Stuttgart, 70569 Stuttgart, Germany; ‡ Institute for Artificial Intelligence, 9149 University of Stuttgart, 70569 Stuttgart, Germany

## Abstract

Identifying transition states (TSs), the high-energy configurations that molecules pass through during chemical reactions, is essential for understanding and designing chemical processes. However, accurately and efficiently identifying these states remains one of the most challenging problems in computational chemistry. In this work, we introduce a new generative AI approach that improves the quality of initial guesses for TS structures. Our method can be combined with a variety of existing techniques, including both machine-learning models and fast, approximate quantum methods, to refine their predictions and bring them closer to chemically accurate results. Applied to TS guesses from a state-of-the-art machine-learning model, our approach reduces the median structural error to 0.077 Å and lowers the median absolute error in reaction barrier heights to 0.40 kcal mol^–1^. When starting from a widely used tight-binding approximation, it increases the success rate of locating valid TSs by 41% and speeds up high-level quantum optimization by a factor of 3. By making TS searches more accurate, robust, and efficient, this method could accelerate reaction mechanism discovery and support the development of new materials, catalysts, and pharmaceuticals.

## Graphical abstract

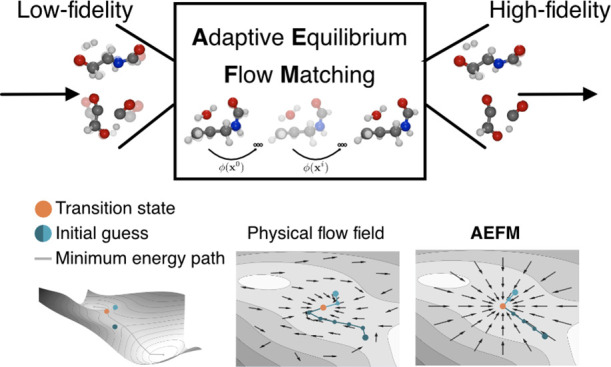

## Introduction

The transition state (TS) plays a central role in elucidating reaction mechanisms and understanding the microkinetic behavior of chemical processes.^1–7^ A detailed knowledge of the underlying kinetics enables the rational design of catalysts, synthetic routes, and functional materials, driving progress toward more efficient, sustainable, and innovative chemical processes.^8,9^ Computationally, a TS corresponds to a first-order critical point on the potential energy surface (PES). Traditional molecular modeling algorithms for locating TSs fall into two broad categories, single-ended^10–12^ and double-ended methods.^13–15^ Single-ended methods refine an initial 3D structure using gradient and sometimes Hessian information, while double-ended approaches construct a continuous path between reactant and product geometries to locate the TS along this path. However, when based on high-level electronic structure methods such as density functional theory (DFT),^16^ these algorithms require substantial computational resources, posing a significant bottleneck in reaction-mechanism discovery.

To overcome this limitation, machine learning (ML) has emerged as a promising direction. Surrogate models, such as Gaussian process regressions^17–23^ or machine-learned interatomic potentials (MLIPs),^24–30^ can approximate the PES, significantly accelerating TS searches when coupled with traditional optimization schemes. However, these approaches require high-quality nonequilibrium data, particularly around the TS region, which limits their scalability.^28^ Recently, Zhao et al. systematically benchmarked and analyzed different training strategies and model formulations for using MLIPs in TS search, highlighting both their current potential and the remaining challenges related to generalization and robustness.^29^ Beyond surrogate-assisted optimization, other deep learning approaches aim to directly predict the TS structure.^31–34^ Many of these methods predict the TS distance matrix and then convert it into 3D coordinates. More recently, generative models have reframed TS prediction as a distribution learning problem, aiming to learn the distribution of TS geometries conditioned on given reactant and product structures.^35–40^ For instance, OA-ReactDiff^36^ models the joint distribution of reactant, TS, and product using denoising diffusion and inpainting to sample plausible TS candidates. Its successor, React-OT,^39^ leverages flow matching (FM)^41,42^ and optimal transport to improve generation accuracy and efficiency. Other models bypass the need for 3D input entirely by generating TS geometries directly from 2D molecular graphs.^37,38^ While generative models are promising, they can struggle to resolve fine-grained geometric details, sometimes producing unphysical features such as atomic collisions or distorted bond lengths.^38,43–46^ In contrast, TS guesses from approximate quantum chemical methods, such as tight-binding, are generally physically reasonable, but can deviate unpredictably from DFT-level structures.^47^ In both scenarios, the predicted TS structures function as low-fidelity approximations that, while providing valuable initial estimates for reaction exploration, may require further refinement to achieve the accuracy needed for quantitatively reliable kinetic analysis.

To address this gap, we introduce Adaptive Equilibrium Flow Matching (AEFM), a structure-only refinement method that transforms low-fidelity TS guesses, regardless of their origin, into high-accuracy TS geometries without requiring any energy or gradient information. AEFM learns to invert noise-injected perturbations of reference TS structures using a novel time-independent form of variational flow matching (VFM).^48^ The model operates by predicting integration steps that iteratively refine the structure, converging toward a fixed-point solution. By additionally respecting the symmetry inherent in molecular structures, AEFM introduces a SE(3)-equivariant method that facilitates robust inference, adaptable to the quality of the initial TS structure. To further improve the chemical realism of refined structures, we incorporate a physics-inspired bond-based loss that guides the model toward physically plausible geometries. AEFM is particularly suited for high-throughput settings, where efficient and reliable refinement is essential to handle large numbers of candidates. Additionally, it benefits in-depth mechanistic studies by reducing the need for costly TS optimization steps. When combined with React-OT, an ML-based model, AEFM reduces the median root-mean-square deviation (RMSD) of predicted TS structures from 0.092 to 0.088 Å, and lowers the median absolute error in barrier heights from 1.092 to 0.793 kcal mol^–1^, representing a 27% improvement over React-OT alone. Incorporating a physics-inspired bond-length loss improves structural realism, as the bonded distance distribution of AEFM-refined samples aligns more closely with the ground truth from the Transition1x data set,^49^ with the Wasserstein-1 distance decreasing from 0.0023 to 0.0015 Å. AEFM also boosts the chemical validity rate of GFN2-xTB-generated^50^ TSs by 41%, making the combined approach a practical solution for rapid and accurate TS discovery. Furthermore, the transferability of AEFM is demonstrated on three out-of-distribution benchmark sets comprising nearly 600 TS structures and their corresponding low-fidelity guesses, where AEFM reduced the median energetic error by up to 75%, corresponding to an absolute reduction of up to 27 kcal mol^–1^.

## Methods

### Overview

AEFM builds on the principle of FM, which learns to transform samples from one distribution into another. The transformation is done by learning a time-dependent vector field that transports samples from the prior distribution to the target distribution along a predefined probability path. In our case, as illustrated in Figure 1, the goal is to refine low-fidelity TS structures, such as those predicted by ML models or semiempirical methods, into high-quality TS geometries. To train AEFM, we start from accurate TS structures taken from a reference data set, which define the target distribution. To reflect the kind of inaccuracy expected from low-fidelity initial guesses, we perturb the reference structures with noise proportional to the prior method’s typical error. This scaling allows AEFM to adapt to the error magnitude of the input source, enabling it to generalize across different prior methods. AEFM then learns a continuous transformation, guided by optimal transport, that maps these noisy inputs back to their original high-fidelity TS geometries. At inference time, however, the quality of low-fidelity TS samples varies, as some may already lie close to the desired distribution, while others deviate significantly. A standard FM inference scheme would apply a uniform integration of the velocity field across all inputs, which can lead to under- or overshooting depending on the initial error. To address this, we instead train AEFM without a time-dependent formulation, resulting in a time-independent equilibrium flow field. This field consistently points toward the high-fidelity structures, enabling fixed-point inference that iteratively pulls each sample toward its refined geometry, regardless of its initial deviation. The number of refinement steps adapts dynamically to the quality of the input, allowing the model to allocate computational effort where it is most needed. To respect molecular symmetries, such as rotation, translation, and atom index permutation, we employ the SE(3)-equivariant LEFTNet^51^ architecture as the backbone of our model. In addition, we propose to incorporate domain knowledge through a bond-loss term, which encourages physically plausible predictions by penalizing unrealistic bond configurations. This explicit inclusion of physics-based constraints guides the model toward more chemically meaningful outputs.

**Figure 1:**
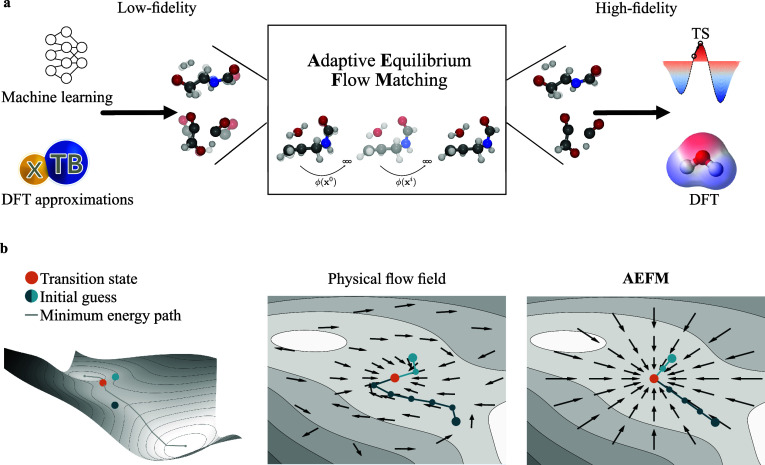
AEFM pipeline for TS structure refinement. (a) The input consists of low-fidelity TS samples, which may originate from various sources such as ML models or tight-binding approximations. These inputs are iteratively refined to produce high-fidelity, chemically valid TS geometries near the DFT level. (b) Comparison between actual physical flow and the one learned by AEFM on the Müller–Brown potential energy surface. Integrating the physical flow field requires multiple function evaluations, which can become computationally expensive with methods such as DFT. In contrast, AEFM learns a much simpler representation that captures the essential structure while requiring significantly fewer and more efficient evaluations.

**Figure 2:**
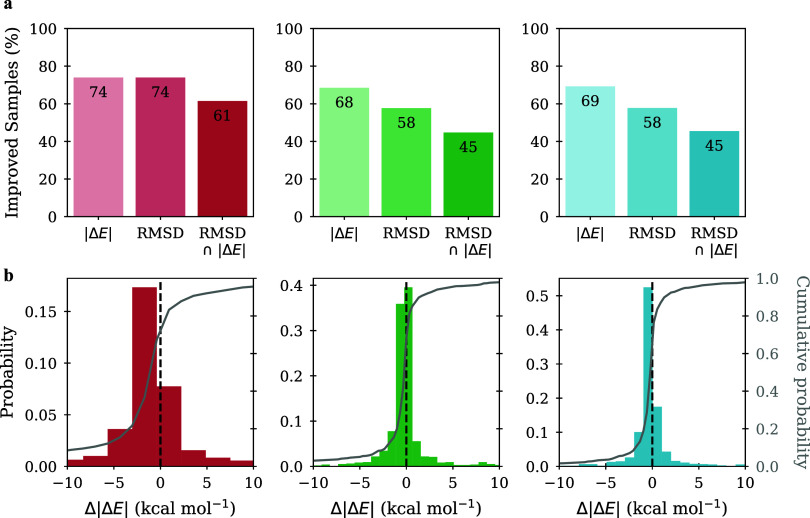
Performance summary of AEFM across diverse low-fidelity sources. (a) Percentage of test samples showing improvement in energy difference |Δ*E*| relative to the reference TS (irrespective of RMSD), in RMSD (irrespective of energy), and in both RMSD and energy difference (RMSD ∩ |Δ*E*|). (b) Histogram (colored, left *y*-axis) and cumulative distribution (gray, right *y*-axis) of the change in energy difference between the low-fidelity and AEFM fine-tuned samples, measured relative to the reference TS. Negative |Δ*E*| values indicate that the refined samples are energetically improved.

**Figure 3:**
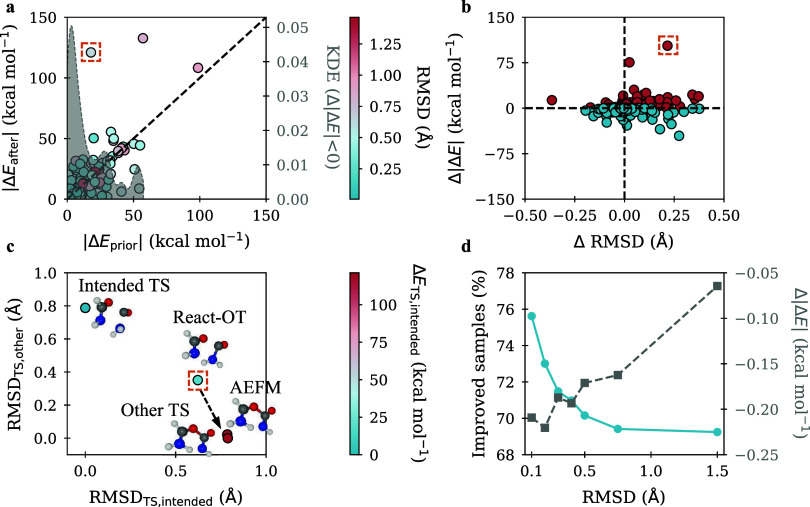
Relationship between energetic and structural changes in AEFM refinements, with a focus on outliers and correlation trends. (a) Energetic differences of AEFM-refined structures vs initial React-OT predictions on the left *y*-axis. Points below the diagonal line indicate improved agreement with the reference TS, while points above reflect increased deviation. On the right *y*-axis, the KDE of energetic improvement weighted by the improvement magnitude is shown. Additionally, an outlier (top left) shows a nearly 6-fold increase in error after fine-tuning. (b) Energetic vs geometric changes resulting from the application of AEFM. The bottom-left quadrant indicates improvements in both structural and energetic similarity, while the bottom-right quadrant reflects improved energy alignment accompanied by reduced structural similarity. Blue points indicate an energetic improvement, while red points correspond to increased dissimilarity. (c) Structural analysis of the outlier. The *x*-axis shows RMSD to the intended TS, and the *y*-axis shows RMSD to an alternative, structurally similar TS. Displayed are the initial React-OT prediction, the fine-tuned sample, and both TS structures. (d) Improvement rate (left *y*-axis in blue) and mean reduction in energy error (right *y*-axis in gray) as a function of the initial React-OT RMSD.

AEFM introduces several methodological innovations to enable efficient and accurate refinement of TS structures. Unlike standard FM, which relies on a time-dependent vector field and fixed integration schedules, AEFM learns a time-independent equilibrium flow field that supports adaptive fixed-point inference. To promote chemically realistic outputs, a physics-inspired bond-length loss that penalizes implausible bond distortions is incorporated. Together, these design choices enable a structure-only training and inference pipeline, eliminating the need for potential energy surface evaluations or gradient computations. As a result, AEFM achieves up to a 40% reduction in mean barrier height errors within just four inference steps, typically completing in less than a second. When quantum mechanical optimization is still required, AEFM-refined structures lead to a 3-fold reduction in computation time compared to unrefined inputs, substantially lowering computational cost while improving stability.

### Flow Matching

FM^41,42^ is a generative modeling approach that learns a transformation from a simple base distribution *q*_0_ to a target distribution *q*_1_. The base distribution *q*_0_ is often referred to as the prior, and the target distribution *q*_1_ as the data distribution.

To model this transformation, FM learns a continuous-time vector field **v**_θ_(**x**_*t*_, *t*). The point **x**_*t*_ lies along a predefined interpolation path between samples **x**_0_ ∼ *q*_0_ and **x**_1_ ∼ *q*_1_. Therefore, an optimal transport probability path^52^ with the interpolation variable *t* ∈ [0, 1] is defined as shown in eq 1.

$$p_{t}(x\;|\;x_{0},x_{1})=N(x\;|\;(1-t)x_{0}+tx_{1},\sigma_{\mathrm{FM}}^{2}I)$$ 1

leading to samples given by eq 2:

$$x_{t}=(1-t)x_{0}+tx_{1}+\sigma_{\mathrm{FM}}\epsilon$$ 2

The value of σ_FM_ was set to 0.05 following a hyperparameter search. The corresponding target velocity field is defined in eq 3:

$$v_{t}(x_{t};\;x_{0},x_{1})=\frac{dx_{t}}{dt}=x_{1}-x_{0}$$ 3

In doing so, FM describes how probability mass moves over time from the prior to the data distribution. To train the vector field **v**_θ_, the squared error between the predicted velocity and the target velocity is minimized. The training objective, known as the FM loss, is given by eq 4.

$$L_{\mathrm{FM}}=E_{x_{0},x_{1},t}[∥v_{\theta }(x_{t},t)-(x_{1}-x_{0}){∥}^{2}]$$ 4

As an alternative loss formulation, the model ϕ_θ_(**x**_*t*_, *t*) can be trained to directly predict **x**_1_ at time *t* instead of the velocity, a strategy that has demonstrated improved performance in practice.^53^ This approach is commonly referred to as variational flow matching (VFM).^48^ Once ϕ_θ_(**x**_*t*_, *t*) is trained, new samples can be generated by solving the ordinary differential equation (ODE) in eq 5 forward in time.

$$\;\begin{array}{cc} \frac{dx_{t}}{dt}=\frac{\phi_{\theta }(x_{t},t)-x_{t}}{1-t}, & x_{0}∼q_{0} \end{array}$$ 5

This integration starts from a sample **x**_0_ drawn from the prior *q*_0_, and produces a sample **x**_1_ ∼ *q*_1_ at time *t* = 1, using any black-box ODE solver. Later, we will make ϕ_θ_(**x**_*t*_, *t*) time-independent and use it to iteratively refine approximate solutions **x** ^*k*+1^ = ϕ_θ_(**x** ^*k*^).

### Adaptive Equilibrium Flow Matching

#### Training

A central component of AEFM is its adaptive behavior, which arises from the formulation of the source distribution *p*_0_ that we learn to map to the target distribution *p*_1_. In our case, the target distribution is determined by the high-fidelity TSs from the Transition1x data set.^49^ Given a sample **x**_1_ ∼ *p*_1_, we define the corresponding source sample **x**_0_ ∼ *p*_0_ as a noisy perturbation of **x**_1_ in eq 6.

$$\begin{array}{cc} x_{0}=x_{1}+\sigma \epsilon , & \epsilon ∼N(0,I) \end{array}$$ 6

The key parameter in this formulation is σ, which controls the extent to which the source distribution deviates from the target. We assume that the deviation of low-fidelity samples **x**_1_^w^ from their corresponding high-fidelity TSs can be modeled as Gaussian noise.

Under this assumption, we want to determine the noise scale σ such that the expected error from a Gaussian corruption process with variance σ^2^ matches the expected error between low-fidelity and reference TSs. Specifically, we impose the condition in eq 7,

$$\begin{array}{rl} \underset{\begin{array}{c} x_{1}∼D,\\ x_{0}∼N(x_{1},\sigma^{2}I) \end{array}}{E}\left[\frac{∥x_{0}-x_{1}{∥}^{2}}{N(x_{1})}\right]=\underset{\begin{array}{c} (x_{1},x_{1}^{w})∼D \end{array}}{E}\left[\frac{∥x_{1}^{w}-x_{1}{∥}^{2}}{N(x_{1})}\right] & \end{array}$$ 7

where *N*(**x**_1_) is the number of atoms involved. Since **x**_0_ = **x**_1_ – σ **ϵ**, the left-hand side simplifies, as shown in eq 8, to

$$E_{\epsilon }\left[\frac{∥\sigma \epsilon {∥}^{2}}{N(\epsilon )}\right]=\frac{\sigma^{2}}{N(\epsilon )}\cdot 3N(\epsilon )=3\sigma^{2}$$ 8

Solving for σ, we obtain the expression in eq 9,

$$\sigma ={\left(E_{(x_{1},x_{1}^{w})∼D}\left[\frac{∥x_{1}-x_{1}^{w}{∥}^{2}}{3N(x_{1})}\right]\right)}^{1/2}$$ 9

Thus, σ can be calculated using the mean RMSD of the low-fidelity samples. The source distribution in our setup is designed to model the expected deviation of the low-fidelity predictions from the reference TS. It captures the distribution of typical errors observed in the low-fidelity method and provides a learning signal during training. However, in contrast to the standard FM framework, we do not sample from the prior during inference. Instead, we start from the actual output of the low-fidelity model. As a result, during training the model learns from the prior, represented by noisy TS structures from the Transition1x data set, but at inference it must adapt to the specific errors present in each low-fidelity input. These errors can vary considerably, with some samples being very close to the true TS and others deviating more. Assigning a uniform time value of *t* = 0 to all such samples during inference, as done in conventional FM, may lead to over- or under-correction by the model. To address this, we remove explicit time conditioning during training, allowing the model to implicitly infer the quality of a given input **x**_*t*_. This helps the model estimate how far each sample is from the final prediction target. In practice, this behavior is encouraged through the use of a direct **x**_1_-prediction loss, as described earlier and shown in eq 10, while omitting time as an input to the network.

$$L_{\mathrm{AEFM}}=E_{x_{0},x_{1},t}[∥x_{1}-\phi_{\theta }(x_{t}){∥}^{2}]$$ 10

#### Inference

Since we omit the concept of time, we no longer integrate the ODE from eq 5. Instead, we train a neural network ϕ_θ_ to directly predict the end point **x**_1_ of a dynamical process starting from an initial point **x**_0_. This formulation aligns with the perspective of VFM, where learning a velocity field that matches trajectories between **x**_0_ and **x**_1_ can be reinterpreted as minimizing a divergence between model and reference end point distributions. In our case, although we do not instantiate or evaluate **v**_θ_(**x**_*t*_, *t*) directly at test time, the network’s prediction implicitly corresponds to the result of integrating such a field over time. In this sense, our model acts as a learned approximation of the ODE solution operator. To further refine predictions and ensure consistency with underlying dynamics, we employ a fixed-point iteration scheme at inference time defined by eq 11:

$$x^{k+1}=\phi_{\theta }(x^{k})$$ 11

where the initial guess **x** ^0^ is taken as the low-fidelity prediction, **x**_1_^w^. Conceptually, this mirrors the inference procedure in Deep Equilibrium Models,^54,55^ where a neural network is iterated to convergence at test time to find a fixed point x* satisfying x* = *f*_θ_(x*). To perform the iteration, one may employ any fixed-point solver, such as Broyden’s method^56^ or Anderson acceleration.^57^ In this work, we use the latter, which enhances convergence by leveraging multiple previous iterates and their residuals to extrapolate a more accurate fixed point. Given *m* previous iterates **x** ^*k*–*m*^, ···, **x** ^*k*^ and corresponding residuals **g**(**x** ^*i*^) = ϕ_θ_(**x** ^*i*^) – **x** ^*i*^, the method solves a least-squares problem to find coefficients **α** such that the weighted sum of residuals ∑_*i* = 0_^*m*^α_*i*_ **g**(**x** ^*k*–*m*+*i*^) is minizimed. Given **α**, the next iterate is computed as shown in eq 12:

$$x^{k+1}=\beta \sum_{i=0}^{m}\alpha_{i}\phi_{\theta }(x^{k-m+i})+(1-\beta )\sum_{i=0}^{m}\alpha_{i}x^{k-m+i}$$ 12

where β ∈ [0, 1] is a damping parameter and ∑_*i*_α_*i*_ = 1. The fixed-point iteration is terminated once the RMSD between successive iterates falls at or below a threshold of 0.01, as defined in eq 13:

$$\frac{∥x^{k+1}-x^{k}∥}{\sqrt{N(x^{k})}}\leq 0.01$$ 13

If the convergence criterion is not satisfied, inference is terminated after a maximum of 100 iterations. The damping parameter β is set to 1.0 and the history size *m* to 5, based on a hyperparameter search.

### Physical Consistency Loss

To address issues such as bond length inconsistencies and atomic clashes in generative models, we introduce an additional loss term focused on bonding.^38,43–46^ This is particularly important because the PES is highly sensitive to small geometric deviations. In some cases, accurately reproducing critical bond lengths is more important than minimizing the overall positional error. A prediction may yield a low RMSD while still introducing small but chemically significant distortions in key bonds, resulting in large energetic errors. To improve the chemical plausibility of generated structures, we compare the local environment of each atom within a cutoff radius *r*_cut_ to that of the corresponding atom in the ground truth structure, as shown in Figure 4b and eqs 14 and 15.

$$L_{b}=E\left[\sum_{\left(i,j)\in B(x_{1}\right)}\frac{[d_{ij}(\phi_{\theta }(x_{t}))-d_{ij}(x_{1}){]}^{2}}{\left|B(x_{1})\right|}\right]$$ 14

$$B(x_{1}):=\{(i,j)|∥x_{1,i}-x_{1,j}∥<r_{\mathrm{cut}}\}$$ 15

with *d*_*ij*_ = ∥**x**_*i*_ – **x**_*j*_∥ as the Euclidian distance between atom *i* and *j*. The cutoff radius is set to 2 Å, based on the longest equilibrium bond lengths typically observed in C, N, O, and H chemistry, with an added margin to accommodate extended bond distances that may arise in TS structures.^58^ Thus, the total loss used in training is shown in eq 16:

$$L=L_{\mathrm{AEFM}}+w_{b}L_{b}$$ 16

with *w*_b_ as a hyperparameter to weight the bond loss influence during training, which we fix to 1.0.

**Figure 4:**
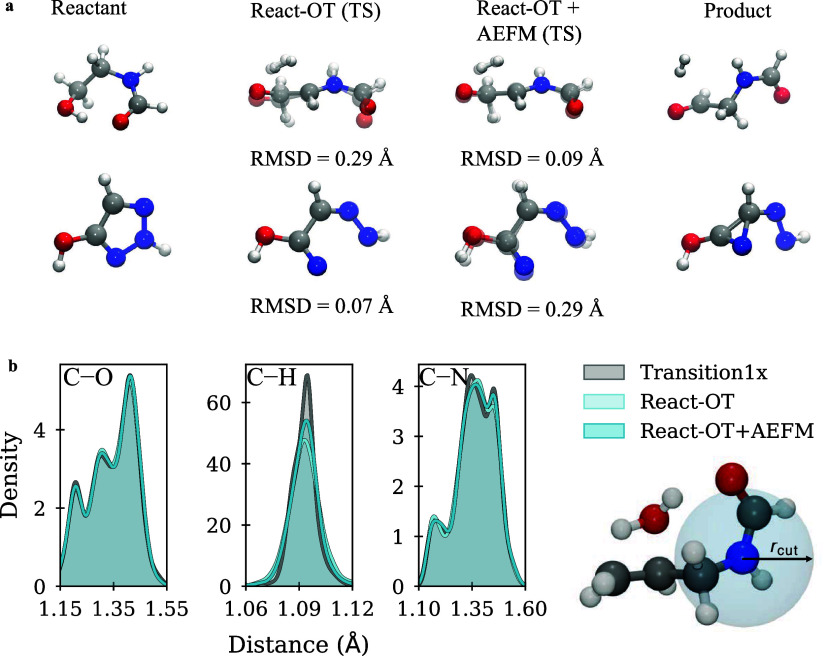
Structural refinement by AEFM. (a) Visual examples of AEFM-based refinement for different initial TS guesses produced by React-OT. The predicted TS is overlaid transparently on the reference TS from the Transition1x data set. (b) Distributions of C–H, C–N, and N–O bond lengths in the Transition1x data set compared to those in the React-OT and AEFM-refined structures.

### Quantum Chemical Validation

To compute the electronic energy of samples, we use ORCA5.0.4 ^59^ in combination with ASE^60^ at the same level of theory as the Transition1x data set^49^ was generated with ωB97*x*/6–31G­(d).^61,62^ To generate the GFN2-xTB^50^ TS guesses, CI-NEB^14^ with ASE and the python interface tblite is utilized. For the CI-NEB computations, the same protocol is used as for Transition1x generation. The NEB calculation is first run until the maximum force perpendicular to the path falls below a threshold of 0.5 eV Å^–1^. Subsequently, the CI-NEB refinement continues until convergence, defined as a maximum perpendicular force below 0.05 eV Å^–1^ or a maximum of 500 iterations. Reactions that do not meet this criterion are considered not converged. For TS optimization, the Sella package^63^ using the P-RFO^10,11^ algorithm along with the ASE ORCA calculator is run until the maximum force of 0.001 eV Å^–1^ is achieved with a maximum number of 300 iterations. It should be noted that the CI-NEB structures from the Transition1x data set, while approximating the TS, still require a median of 15 p-RFO optimization steps to satisfy this convergence criterion (Figure 5c). Numerical Hessians are computed using finite central difference method with an δ of 0.01 Å. The intrinsic reaction coordinate (IRC) calculations were performed using the Sella package with a maximum of 500 iterations, and the resulting minima were further relaxed using the BFGS algorithm until the maximum force fell below 0.05 eV Å^–1^.

**Figure 5:**
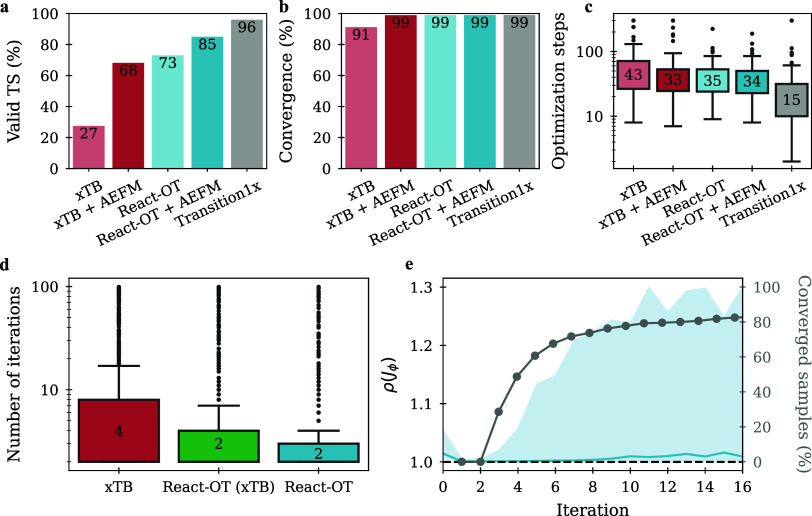
Chemical validation and fixed-point convergence analysis. (a) Fraction of valid TS structures, defined by the presence of exactly one imaginary frequency in the Hessian. (b) Convergence rate of DFT TS optimizations. (c) Boxplot of DFT optimization steps required to reach a converged TS structure. (d) Number of iterations required by AEFM to reach a fixed point. Convergence is defined by an RMSD below 0.01 between successive iterates; otherwise, inference is terminated after 100 iterations. (e) Spectral radius of the model’s Jacobian with respect to the input structure, shown as median (solid line) and interquartile range (shaded region) over iterations (left *y*-axis). The percentage of converged samples is plotted on the right *y*-axis. Contractive behavior ensuring convergence occurs when ρ­(*J*_ϕ_) < 1.0, while advanced solvers still succeed beyond this threshold.

## Results

### Refining TS Structures Across Fidelity Scales

To evaluate AEFM, we use the Transition1x data set,^49^ which contains climbing-image nudged elastic band (CI-NEB)^14^ calculations performed with DFT (ωB97*x*/6–31G­(d)^61,62^ ) for 10,073 organic reactions encompassing diverse reaction types. These reactions were sampled from an enumeration of 1154 reactants in the GDB7 data set,^64^ which includes molecules with up to 7 heavy atoms (C, N, and O) and a total of 23 atoms. We adopt the same random split as Duan et al.,^36^ using 9000 reactions for training and 1073 for testing.

AEFM is applied to refine prior low-fidelity TS structures toward valid TS geometries at the target level of theory. To assess the quality of the refined structures, we evaluate both the RMSD of atomic positions and the absolute error in the reaction barrier.

To assess the effectiveness of AEFM, we consider React-OT^39^ as the first low-fidelity source, a state-of-the-art generative model for TS prediction. React-OT achieves remarkable accuracy, producing samples with a mean RMSD of 0.18 Å and a median absolute error in barrier height of 1.092 kcal mol^–1^. Applying AEFM to refine the React-OT samples yields a 27% improvement in the median barrier height error, requiring only 2 model calls in median and approximately 0.13 s per refinement on an Nvidia A40 GPU. Consequently, 69% of the TSs had a more accurate barrier height, achieving a median absolute error of 0.793 kcal mol^–1^. We further evaluated AEFM by substituting the LEFTNet backbone with an out-of-the-box EquiformerV2 ^65^ model. This combination reduced the median barrier height error to 0.402 kcal mol^–1^, corresponding to a 63% improvement over the original React-OT samples.

As a second low-fidelity source, we consider GFN2-xTB,^50^ a tight-binding approximation applicable across a broad range of chemical systems and therefore a widely adopted starting point for elucidating reaction mechanisms. Tight-binding methods are approximately 3 orders of magnitude faster than DFT, enabling high-throughput reaction scans that would be otherwise computationally prohibitive. For the 1073 test reactions, reactant and product geometries were first relaxed, followed by CI-NEB calculations using GFN2-xTB. Of these, 945 calculations converged successfully, yielding samples with a mean RMSD of 0.31 Å and a median absolute error in barrier height of 2.673 kcal mol^–1^. Applying AEFM improves the median absolute error in barrier height by 59%, reducing it to 1.090 kcal mol^–1^, while requiring only a median of 4 model calls. To contextualize this improvement, in microkinetic modeling, an error of 1 order of magnitude change in reaction rate is considered as chemical accuracy, corresponding to 1.58 kcal mol^–1^ error in barrier height at 70 °C.^66,67^ Analyzing the chemical accuracy of samples reveals that only 25% of the original GFN2-xTB-generated structures meet this threshold, whereas AEFM refinement increases this accuracy rate to 57%.

To reduce the computational cost of generating DFT-quality reactant and product structures, we follow Duan et al.^39^ and employ React-OT directly on xTB-optimized geometries. This approach enables rapid TS generation without requiring expensive DFT-level optimization of end points. React-OT can be reliably applied to xTB-level structures, yielding a mean RMSD of 0.21 Å and a median absolute error in barrier height of 1.186 kcal mol^–1^. Building on this, we apply AEFM to further refine the resulting TS guesses, reducing the median absolute error to 0.824 kcal mol^–1^, corresponding to a 31% improvement, with only a median of two model evaluations. The results of AEFM applied to each low-fidelity method are summarized in Table 1.

**Table 1:** Performance of AEFM Refinement^a^

	RMSD (Å)		|Δ*E*_TS_| (kcal mol^–1^)		
approach	mean	median	mean	median	inference (s)
xTB CI-NEB	0.312	0.179	10.426	2.673	9.23
xTB CI-NEB + AEFM	0.250 (↓20%)	0.119 (↓34%)	6.204 (↓40%)	1.090 (↓59%)	+0.24
xTB CI-NEB + AEFM^b^	0.259 (↓17%)	0.097 (↓46%)	7.370 (↓29%)	0.572 (↓79%)	+0.24
React-OT (xTB)	0.211	0.108	4.697	1.186	0.14
React-OT (xTB) + AEFM	0.214 (↑1%)	0.102 (↓6%)	4.153 (↓12%)	0.824 (↓31%)	+0.12
React-OT (xTB) + AEFM^b^	0.208 (↓1%)	0.090 (↓17%)	4.425 (↓6%)	0.455 (↓61%)	+0.12
React-OT	0.183	0.092	3.405	1.092	0.14
React-OT + AEFM	0.188 (↑3%)	0.088 (↓4%)	3.341 (↓2%)	0.793 (↓27%)	+0.13
React-OT + AEFM^c^	0.176 (↓4%)	0.086 (↓7%)	3.158 (↓7%)	0.790 (↓27%)	+0.13
React-OT + AEFM^b^	0.179 (↓4%)	0.077 (↓17%)	2.886 (↓15%)	0.402 (↓63%)	+0.13

a Structural and energetic errors of various low-fidelity TS guesses before and after refinement with AEFM. The refinement consistently reduces both mean and median deviations relative to the reference TS structures. In addition, average inference times per sample are reported, showing that AEFM introduces only negligible computational overhead.

b Results obtained using an out-of-the-box EquiformerV2 model.

c For 26 reactions, a different intended TS was selected if the RMSD between the low-fidelity sample and this alternative TS was at least 30% lower than the RMSD to the originally intended TS.

While the absolute improvements for React-OT samples on Transition1x appear modest, this is largely because ReactOT is trained on this data set and its TS guesses are already close to the reference structures, leaving little room for further refinement. The true strength of AEFM becomes apparent when applied to more challenging scenarios or alternative priors. To demonstrate this, we evaluated AEFM on three external benchmark sets that provide TS guesses, where it consistently achieves substantial improvements and showcases its ability to refine structures under diverse and previously unseen conditions. The first contains 15 Diels–Alder TSs generated at the PM6 level of theory,^68^ and the second comprises 500 KinBot-generated TS guesses for organic reactions.^63^ All structures were refined using P-RFO optimization at the ωB97*x*/6–31G­(d) level to obtain reference geometries. Across both data sets, AEFM consistently reduced the median energetic error, in some cases by up to 75%, or up to 27 kcal mol^–1^ in absolute terms. The third benchmark consists of 402 CI-NEB TSs for a ruthenium-catalyzed ethylene hydrogenation reaction,^69^ computed using the same workflow as Transition1x at the B3LYP/def2-SVP level of theory. From this set, 295 structures were used to fine-tune a pretrained AEFM model, and the remaining low-fidelity test guesses were generated using CI-NEB with GFN2-xTB. Despite the substantially larger molecular sizes, with a median of 90 atoms, AEFM again delivered strong improvements over the initial structures, highlighting its ability to handle transition-metal systems and its suitability for realistic catalytic applications. A detailed quantitative summary, including RMSD and energetic statistics, is provided in Supplementary Table S9.

### Understanding Refinement Dynamics

To further investigate the performance of AEFM, we conduct a detailed analysis across diverse scenarios, aiming to better understand the factors influencing its strengths and limitations. A first aspect we examine is the asymmetry in the distribution of barrier height errors, which is particularly evident for refined samples generated using React-OT as prior. Figure 3a shows pre- and postrefinement energetic errors, where points below the bisecting line indicate improvement. The second axis represents a kernel density estimate of the energetic improvement plotted against the initial energy deviation. To highlight where AEFM is most effective, the density is additionally weighted by the magnitude of improvement. This provides insight into which samples, characterized by their initial energetic difference, benefit the most from refinement. An illustrative outlier contributing to the skewed mean is shown in Figure 3c. For the particular reaction, we consider four TS, the reference (intended) TS, the React-OT prediction, its fine-tuned version obtained via AEFM, and an alternative TS associated with a different but structurally similar reaction. The plot illustrates the structural deviation, measured as RMSD, to the intended TS on the *y*-axis and to the alternative TS on the *x*-axis, while the marker color encodes the relative energy with respect to the intended TS. The original React-OT prediction deviates notably from the intended TS, with an RMSD of 0.632 Å and an energy difference of 17.904 kcal mol^–1^. After fine-tuning, the sample shifts further away from the intended TS, reaching an RMSD of 0.793 Å and a significantly larger energy difference of 120.993 kcal mol^–1^. At first glance, this might appear to be a failure of the optimization process. However, comparison with the alternative TS reveals a different picture, the fine-tuned structure is nearly identical to this other TS, exhibiting an RMSD of just 0.048 Å and an energy deviation of merely 0.256 kcal mol^–1^. This behavior is explained by the initial proximity of the React-OT sample to the alternative TS, with an RMSD of 0.359 Å compared to the intended TS. Since AEFM operates purely on structural refinement and is trained on perturbed TS geometries without access to reactant-product context, it interprets the input as a noisy version of the alternative TS and converges accordingly. To further analyze this effect, all React-OT samples were compared with similar other TS. To ensure that the alternative TSs are meaningfully closer to the sample, we only retain cases in which the RMSD to the alternative TS is at least 30% lower than the RMSD to the originally intended TS. The mean RMSD is now improved by 7% and the absolute energetic error by 5% compared to the initial analysis of fine-tuned samples. This example highlights an essential characteristic of the approach, in the absence of explicit reaction context, AEFM fine-tunes samples toward structurally and energetically valid TSs, which may not always correspond to the originally intended reaction. Such behavior is typical for surface walking algorithms, where the target is to find any nearby viable TS given an initial guess structure.^10–12^

A key element influencing the performance of AEFM is the quality of the initial guess. Figure 3d illustrates this by showing the percentage of energetically improved samples along the left *y*-axis, and the corresponding mean energy improvement along the right *y*-axis, both plotted against increasing RMSD thresholds applied to the initial React-OT samples. At each threshold, only those samples with an initial RMSD below the given value are included in the statistics. The results show a clear trend, with both the likelihood and magnitude of improvement being higher at lower RMSD thresholds. Specifically, for samples with RMSD below 0.2 Å, 73% of the reactions show an energetic improvement after fine-tuning, with a mean improvement of 0.15 kcal mol^–1^. In contrast, at higher thresholds, we have 69% improved reactions and a mean energetic improvement of 0.06 kcal mol^–1^. To visually illustrate the behavior of AEFM across different initial TS guess qualities, Figure 4a presents representative examples of initial React-OT predictions and their AEFM-refined structures. The first example depicts the oxidation of an alcohol to an aldehyde with simultaneous hydrogen release. The initial React-OT TS guess struggles with the positioning of the dissociating molecular hydrogen and the terminal aldehyde group, for which the carbon oxygen bond length is predicted to be too short (1.282 Å instead of 1.297 Å). These inaccuracies lead to an initial RMSD of 0.29. The second example shows the ring-opening of a hydroxy-triazole, for which React-OT predicts the TS with high accuracy (RMSD 0.07 Å), whereas AEFM degrades the prediction, resulting in a final RMSD of 0.29 Å. In this case, AEFM overestimates the carbon–carbon bond length to 1.543 Å instead of the reference value of 1.485 Å, and additionally mispredicts the angle involving the oxygen and nitrogen atoms.

A notable feature of AEFM is its rapid and efficient training. Since the model operates on slightly perturbed TS structures, it converges as fast as 600 epochs. In contrast, React-OT involves a more complex training pipeline, consisting of 2000 epochs for training the diffusion model,^36^ followed by 200 additional epochs for the optimal transport loss.^39^ A more detailed comparison is added in the Supporting Information. Beyond training time, AEFM also demonstrates strong data efficiency. To assess this, we evaluated the impact of training set size on fine-tuning CI-NEB xTB samples. As shown in Supplementary Figure S1, increasing the amount of training data systematically reduces the energy error, both in terms of the mean and the median. Notably, using only 4000 training samples, less than half of the whole data set, already achieves a 25% reduction in mean absolute error in barrier height, compared to the 40% reduction obtained using the full 9000 samples.

### Physics-Informed Loss Improves Chemical Validity

In some cases, the potential energy surface is highly sensitive to subtle variations in bond lengths, as certain bonds contribute disproportionately to the total energy due to their stiffness. Standard coordinate-based loss functions, such as mean squared error, apply uniform weighting to atomic displacements, making them ill-suited to capture the varying energetic sensitivities across different degrees of freedom. To address this, AEFM incorporates an additional bond-based loss (see eq 14). During training, the model explicitly compares the chemical environment around each atom within a 2 Å cutoff radius of the ground truth TS to that of the predicted structure. For this purpose, a neighbor list is constructed for each atom in the ground truth TS by identifying all atoms within the cutoff. The same neighbor list is then applied to the predicted structure, and the corresponding interatomic distances are compared to those in the ground truth. By aligning local environments in this way, the model is encouraged to maintain realistic bonding patterns and penalize unphysical distortions, reinforcing chemical consistency and improving energetic fidelity in its predictions.

To assess the impact of the bond loss term, we compare AEFM’s fine-tuning performance when including the term versus omitting it, using two representative low-fidelity sources, React-OT^39^ and GFN2-xTB.^50^ For React-OT samples, incorporating the bond loss results in a 27% reduction in the median absolute error of barrier heights. In contrast, the same model without the bond loss achieves only a 3.5% improvement (Supplementary Table S5). To understand the source of this improvement, we analyze how the bond loss affects the model’s ability to recover chemically plausible local structures. Specifically, we evaluate whether the refined structures better match the local environment within a 2 Å neighborhood of each atom present in the data set. Interactions in this environment are categorized as either bonded or nonbonded based on threshold distances (Supplementary Table S3). With the bond loss, the similarity to the reference bond length distributions improves, with the Wasserstein-1 distance decreasing from 0.0023 to 0.0015 Å for bonded interactions (a 36% improvement) and from 0.0060 to 0.0056 Å for nonbonded interactions (a 6% improvement), as illustrated for selected bonds in Figure 4b. Given that bonded interactions dominate the intramolecular potential energy landscape, enhancing their accuracy is critical for reliable energy predictions. This effect is even more pronounced for xTB samples, which exhibit larger deviations from the target distribution. Here, the bond loss leads to a decrease of the Wasserstein-1 distance from 0.0057 to 0.0025 Å (a 57% improvement) and from 0.0176 to 0.0080 Å (a 54% improvement) for nonbonded ones (Supplementary Table S4).

Moreover, average displacement metrics such as RMSD often fail to reflect meaningful changes in energy, underscoring their limited sensitivity, as shown in Figures 2a and 3b. Notably, the fraction of samples that improve in both RMSD and energy is considerably smaller than the fraction that improve in energy alone (Figure 2a). In line with this, the correlation between energetic and structural improvement is weak, with a Pearson coefficient of only 0.17 (Figure 3b). A similarly weak relationship between RMSD and energy difference was also reported by Duan et al.^36^ That highlights that generating realistic bond lengths in the refinement process is just as crucial as minimizing deviations in atomic positions. In many TS structures, the energetic accuracy is governed primarily by the reactive center. Consequently, even if the RMSD improves slightly for some atoms, introducing unrealistic bonds, such as excessively short ones, can severely degrade energetic similarity.^70^ This effect is further illustrated by the distribution of C–H bond lengths, which, after refinement with AEFM, shows a 44% higher similarity to the data set distribution compared to the original React-OT samples. While the refined C–H bond might not match the exact pose of the reference, its physically accurate length improves energetic similarity, even if the overall RMSD appears worse.

This observation relates to a broader challenge in molecular generative modeling, generating chemically consistent bond geometries.^38,43–46^ Several recent works have proposed solutions to mitigate this issue. For example, Boltz-1 ^46^ biases generation toward low-energy configurations using physically inspired energy functions. While effective in diffusion-based generation schemes, this approach is incompatible with our fixed-point inference method, which does not rely on stochastic sampling. Vost et al.^45^ address the sensitivity of generative models to geometric distortions by augmenting training data with perturbed structures and conditioning the diffusion process on the distortion level. However, this requires training a diffusion model from pure Gaussian noise on distortion-conditioned data, whereas our method uses an adaptive prior. Williams and Inala^44^ propose a physics-informed diffusion model that decomposes the generative task into separate components for bonding, bending, torsion, and chirality, enabling more physically grounded predictions. This decomposition, however, depends on a specialized neural network architecture and limits the flexibility to choose general-purpose backbones. Finally, Falck et al.^71^ analyze the influence of the noising schedule on the recovery of high-frequency features, such as precise bond lengths. While theoretically insightful, their analysis was not conducted in the context of molecular modeling.

These efforts highlight the importance of incorporating structural or energetic priors to improve the physical fidelity of generated molecules. In contrast to more complex solutions, AEFM addresses this issue with a simple yet effective bond loss term, which guides the model toward reproducing the bond distributions found in the underlying data.

### Quantum Chemical Validation

While the combination of tight-binding methods or generative models with AEFM enables fast and robust high-throughput TS screening, a full quantum mechanical treatment remains essential for detailed mechanistic studies.^2,4–7^ In such cases, TSs must be refined using saddle point optimization at the DFT level.^72^ These optimizations typically require multiple evaluations of forces or even full Hessians, making them computationally demanding, even for small molecules.^19,28^ To highlight the practical impact of AEFM on downstream applications, we evaluate its effect on the chemical validity of TS structures and the efficiency of DFT-based TS optimizations. For a representative set of 100 reactions, we compare three key metrics, namely the fraction of valid TS structures (a), identified by exactly one imaginary frequency in the Hessian, the convergence rate of DFT TS optimizations (b), and the number of optimization steps required (c). Each metric is assessed for both the raw input structures and the corresponding AEFM-refined samples. AEFM incurs minimal overhead, mostly requiring only 2 to 5 model evaluations depending on the quality of the initial guess, as seen in Figure 5d. In contrast, full DFT optimizations are significantly more expensive. Applied to GFN2-xTB initial guesses, AEFM increases the fraction of valid TS structures from 27 to 68%, a 41% absolute improvement (Figure 5a). Moreover, AEFM improves the overall convergence rate of TS optimizations from 91 to 99% (Figure 5b), further underscoring its robustness. As a complementary analysis, we further evaluated whether the TSs remain consistent with the intended reactions. To this end, the DFT-refined geometries were compared with the corresponding DFT-refined reference TSs and intrinsic reaction coordinate (IRC) calculations were performed to assess whether the resulting minima match the expected reactant–product connectivity. The results show that a large majority of refined structures remain faithful to the target reaction pathway and yield the correct minima after IRC propagation. A full breakdown and reaction-level statistics are provided in the Supporting Information. Lastly, we analyze the effect of AEFM on the speed of downstream DFT optimization. When using a general prior such as GFN2-xTB, Figure 5c shows that AEFM reduces the median number of DFT optimization steps by 10, corresponding to a 3-fold reduction in CPU hours required for refinement (Supplementary Table S9). However, the speed-up achieved on top of React-OT samples is negligible, reflecting the fact that React-OT already produces high-quality initial TS guesses for Transition1x reactions. These results highlight that while AEFM provides limited additional benefit for specialized high-fidelity priors, it offers a substantial practical advantage when refining lower-quality TS guesses.

### Convergence Analysis

As AEFM relies on fixed-point iteration, understanding its convergence behavior is critical. A standard indicator of local convergence is the Lipschitz constant *L*, which quantifies how sensitively the model output responds to input perturbations. In practice, however, this condition is often evaluated via the spectral radius ρ­(*J*_ϕ_), the largest absolute eigenvalue of the Jacobian *J*_ϕ_ at a given point **x**. By Lyapunov’s linearization theorem, the condition ρ­(*J*_ϕ_) < 1 suffices for convergence in the absence of advanced solvers. However, as Bai et al.^55^ point out, this requirement can be overly conservative in practice. Methods like Broyden’s method^56^ or Anderson acceleration^57^ often succeed even when ρ­(*J*_ϕ_) > 1, due to their ability to handle mild local noncontractive behavior. To assess AEFM’s convergence characteristics, Figure 5e displays the evolution of ρ­(*J*_ϕ_) over refinement iterations on GFN2-xTB samples. Convergence is defined as the point where the RMSD between successive iterates falls below 0.01, as specified in eq 13. If convergence is not achieved, inference is terminated after 100 iterations. The plot displays the median as well as the 25th and 75th percentiles for each iteration, considering only structures that have not converged in earlier steps. As a result, higher iteration numbers are dominated by more slowly converging cases. In addition, the cumulative convergence rate is shown as an overlay. Initially, the spectral radius drops sharply, reflecting strong local contractivity and rapid convergence. After iteration 4, the median convergence point, the spectral radius begins to rise again. This increase does not signal failure but highlights that remaining unconverged samples tend to be more structurally complex and locally less stable. These more complicated cases are pushing the upper quantiles of ρ­(*J*_ϕ_) upward. Still, even in these regions, the 75th percentile remains below 1.3, indicating near-contractive dynamics. Out of 1073 React-OT and 945 xTB samples, only 6 and 3, respectively, failed to converge before reaching the iteration limit. Overall, AEFM achieves fast and stable convergence for the majority of samples, with early iterations characterized by low spectral radii and minimal computational overhead. Although convergence is slower for a few complex cases, they remain computationally manageable, with inference times not exceeding 1.6 s.

## Discussion

AEFM addresses a core challenge in reaction mechanism elucidation by converting low-fidelity TS guesses into chemically accurate, DFT-quality structures with minimal computational cost. By learning a time-independent flow field, conditioned on a prior tailored to the systematic error distribution of approximate methods, AEFM provides reliable refinements across diverse inputs. Each inference call requires only a fraction of a second per structure, enabling seamless integration into high-throughput pipelines without introducing significant computational overhead.

This makes AEFM highly suitable to enhance fast TS generators such as GFN2-xTB or React-OT, improving the chemical viability of their outputs and substantially reducing the effort required for downstream DFT saddle point optimization. This lightweight correction mechanism also opens the door to more complex applications, such as heterogeneous catalysis or enzymatic systems, where initial guesses are costly to refine and subtle structural features are often critical to reactivity.

A central strength of AEFM lies in its use of a physics-informed bond loss, which actively steers refinements toward chemically meaningful local structures, addressing a critical weakness in generative models for molecule generation. Looking ahead, incorporating higher-order geometric features, such as angles and torsions, alongside adaptive interaction cutoffs could unlock even greater accuracy and broaden applicability to more complex chemistries.

Despite its robustness, AEFM is limited by the support of its training prior. For initial guesses that deviate substantially from typical training-time errors, performance degrades. One promising path forward involves a two-stage refinement strategy guided by model uncertainty. A specialized model, trained on broader structural deviations, could be applied when the primary model signals high uncertainty, enabling robust treatment of more strongly perturbed inputs.

Overall, AEFM offers a flexible and computationally efficient paradigm for lifting low-fidelity predictions to chemically and physically meaningful accuracy.

## Supplementary Material



## Data Availability

The Transition1x data set^49^ used in this work is available via Figshare at 10.6084/m9.figshare.19614657.v4. The pretrained AEFM model checkpoints and the corresponding databases for each low-fidelity source are available on Zenodo at 10.5281/zenodo.16414436. The AEFM codebase is publicly available as an open-source repository on GitHub to support continuous development at https://github.com/samirdarouich/AEFM.

## Notes

Comparison to flow matching; ablation studies, including data set size variations, adaptive prior noise scales, physical loss, and the backbone model; illustrative cases of AEFM refinements; in-depth analysis for GFN2-xTB samples; details of the quantum chemical validation and three out-of-distribution benchmarks; and metrics (PDF)
