# Supplementary material for: Adaptive Transition-State Refinement with Learned Equilibrium Flows
Source: J Chem Inf Model. 2026 Feb 2;66(4):2154–65. doi: 10.1021/acs.jcim.5c02902 (PMC12933717; doi:10.1021/acs.jcim.5c02902)
Supplement: Supplementary file 1 [file ci5c02902_si_001.pdf]

Supporting Information

Adaptive Transition State Refinement with Learned Equilibrium Flows

Samir Darouich,[†,‡] Vinh Tong,[‡] Tanja Bien,[‡] Johannes Kästner,[*,†] and Mathias Niepert[*,‡]

†*Institute for Theoretical Chemistry, University of Stuttgart, 70569 Stuttgart, Germany*

‡*Institute for Artificial Intelligence, University of Stuttgart, 70569 Stuttgart, Germany*

E-mail: kaestner@theochem.uni-stuttgart.de; mathias.niepert@ki.uni-stuttgart.de

Comparison to flow matching

An alternative to AEFM is to apply flow matching (FM) using a Gaussian prior and the TSs as the target distribution. The initial time from which the ODE in FM is integrated is inferred based on the mean RMSD between the low-fidelity structures and the reference TSs, using the definition of the intermediate interpolants $\mathbf{x}_t$. Specifically, t_0 is chosen such that the RMSD between the time interpolant $\mathbf{x}_t$ and the target $\mathbf{x}_1$, $\|\mathbf{x}_t - \mathbf{x}_1\|/N$, matches the average RMSD of the low-fidelity source. For GFN2-xTB samples, this yields $t_0 = 0.87$, and for React-OT samples, $t_0 = 0.93$. Supplementary Table S1 reports the corresponding performance. These results are significantly worse than those obtained with AEFM, which can be attributed to the fact that FM must learn the flow field from a Gaussian prior, making the task considerably more complex compared to AEFM. Furthermore, to ensure a

fair comparison with AEFM, the source and target molecules are not aligned, resulting in a non-linear vector field that is harder to integrate and leads to less accurate structures.

Table S1: Performance using FM for refinement.

Approach	RMSD (Å)		$\lvert\Delta E_{\mathrm{TS}}\rvert$ (kcal mol^{-1})	
	Mean	Median	Mean	Median
xTB CI-NEB	0.312	0.179	10.426	2.673
xTB CI-NEB + FM	0.439	0.310	91.732	88.953
React-OT	0.183	0.092	3.405	1.092
React-OT + FM	0.252	0.167	34.723	34.265

Ablation and model architecture

Data size

To evaluate the data efficiency of AEFM, the model was trained using subsets of 2000, 4000, 6000, 8000, and all 9000 training samples. Each trained model was then applied to the GFN2-xTB samples, and the resulting mean and median energetic differences to the ground truth TSs are compared in Supplementary Figure S1. The results reveal a clear trend of decreasing energetic difference with increasing training data, with 4000 samples already providing a substantial improvement in mean energetic difference.

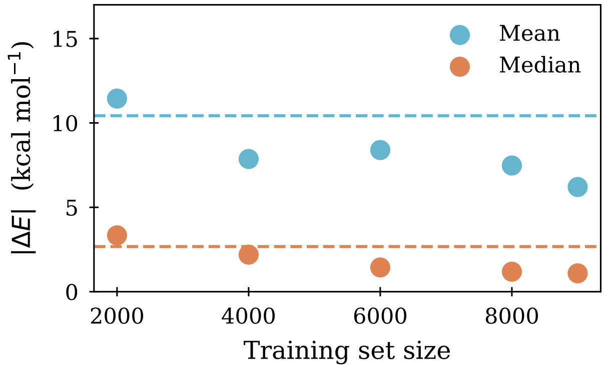

Figure S1: **Data efficiency of AEFM.** Mean and median energy errors for models trained with different sample sizes. Dashed lines show corresponding errors from initial GFN2-xTB samples.

Adaptive prior

To assess the sensitivity of the noise level σ used in the adaptive prior, models trained with different noise settings were evaluated across the various low-fidelity structure sources (Supporting Table S2). When applying AEFM on top of React-OT samples, higher noise levels yield performance comparable to lower noise settings, although they require more optimization steps. In contrast, using a smaller noise level on GFN2-xTB samples leads to a worse mean error but improves the median. This indicates that a lower noise level refines already high-quality geometries, whereas a higher noise level is more effective at correcting larger deviations. Overall, this suggests that the initial noise level can be selected conservatively, but overly small or large values degrade performance. Thus, estimating the noise level based on Equation 9 provides a reliable and practical guideline.

Table S2: Statistical performance of AEFM with different adaptive prior to different samples. Using Equation 9 to estimate the required noise produces $\sigma = 0.11$ for React-OT and $\sigma = 0.19$ for xTB CI-NEB.

Approach	RMSD (Å)		$\|\Delta E_{\mathrm{TS}}\|$ (kcal mol^{-1})		Steps	
	Mean	Median	Mean	Median	Mean	Median
React-OT + AEFM (σ=0.11)	0.188	0.088	3.341	0.793	6	2
React-OT + AEFM (σ=0.19)	0.190	0.084	3.230	0.790	8	2
React-OT + AEFM (σ=0.28)	0.196	0.091	3.958	1.029	11	2
xTB CI-NEB + AEFM (σ=0.11)	0.264	0.132	8.197	1.265	12	3
xTB CI-NEB + AEFM (σ=0.19)	0.250	0.119	6.204	1.090	15	4
xTB CI-NEB + AEFM (σ=0.28)	0.254	0.113	7.042	1.302	15	4

Phyiscal loss

Supplementary Table S4 shows the Wasserstein-1 distance for each bonded interaction using the thresholds defined in Supplementary Table S3. Combining React-OT with AEFM results in consistently lower Wasserstein-1 distances across nearly all bonded and non-bonded interactions, indicating improved agreement with the underlying Transition1x dataset. Omitting the physical loss not only degrades overall model performance (Supplementary Table S5) but

also leads to a markedly poorer reconstruction of the local chemical environment relative to the Transition1x reference structures. This effect is especially pronounced for GFN2-xTB samples, where the bonded similarity improves by just 14% when the bond loss is omitted, compared with a much larger 57% improvement when the bond loss is included. As an additional validation experiment, we tested whether AEFM 's behavior could be explained merely by correcting bond lengths. To do so, we computed the mean bonded distances from the Transition1x dataset and relaxed all ReactOT samples such that their bond lengths matched these averaged values. Instead of improving the structures, this procedure led to a massive deterioration, the RMSD increased to 0.2 Å, and the deviation in energy from the ground-truth TSs rose dramatically to 31.5 kcal mol^{-1}. Additionally, the Wasserstein distance within the 2 Å chemical neighborhood increased by an order of magnitude. These results demonstrate that simply enforcing average bond lengths cannot substitute the behavior learned by AEFM, illustrating that the model captures the underlying distribution of chemically relevant environments rather than applying a trivial geometric correction.

Table S3: Threshold distances used to determine bonded atom pairs. To categorize into bonded and non-bonded, an additional margin of 0.1 Å is added on top of the threshold values.

	C–C	C–H	C–N	C–O	H–H	H–N	H–O	N–N	N–O
Bond Threshold (Å)	1.54	1.09	1.47	1.43	0.74	1.01	0.96	1.45	1.40

Table S4: Wasserstein-1 distance to bond distribution inherent in the test samples of the Transition1x dataset for different bonded (bd) and non-bonded (nbd) atom pairs (lower is better).

Method	C–C	C–H	C–N	C–O	H–H	H–N	H–O	N–N	N–O
xTB CI-NEB (bd)	0.0041	0.0058	0.0097	0.0065	0.0151	0.0046	0.0029	0.0085	0.0113
xTB CI-NEB + AEFM (bd)	0.0032	0.0015	0.0021	0.0018	0.0543	0.0063	0.0140	0.0116	0.0099
React-OT (bd)	0.0022	0.0019	0.0033	0.0019	0.0124	0.0035	0.0059	0.0080	0.0142
React-OT + AEFM (bd)	0.0011	0.0011	0.0023	0.0014	0.0081	0.0034	0.0061	0.0059	0.0110
xTB CI-NEB (nbd)	0.0067	0.0289	0.0192	0.0129	0.0082	0.0461	0.0913	–	0.0697
xTB CI-NEB + AEFM (nbd)	0.0046	0.0097	0.0138	0.0234	0.0027	0.0324	0.0416	0.0981	0.0466
React-OT (nbd)	0.0074	0.0068	0.0128	0.0115	0.0037	0.0088	0.0205	0.1957	0.0413
React-OT + AEFM (nbd)	0.0061	0.0065	0.0106	0.0122	0.0034	0.0139	0.0184	0.0851	0.0518

Table S5: Performance using AEFM without bond loss for refinement.

Approach	RMSD (Å)		$\|\Delta E_{\mathrm{TS}}\|$ (kcal mol^{-1})	
	Mean	Median	Mean	Median
xTB CI-NEB	0.312	0.179	10.426	2.673
xTB CI-NEB + AEFM (w_{b}=0.0)	0.249	0.103	10.405	1.518
React-OT	0.183	0.092	3.405	1.092
React-OT + AEFM (w_{b}=0.0)	0.186	0.083	3.750	1.056

Backbone

The model and training parameters for AEFM are shown in Supplementary Table S7a and Supplementary Table S7b. To test the statistical relevance of the AEFM effect on React-OT samples, the model has been trained with three different seeds and inference was evaluated for all three models as shown in Supplementary Table S6. As an additional benchmark, we trained an out-of-the-box EquiformerV2 model, reserving 1000 of the 9000 training samples for early stopping. The model comprises 76 million parameters and demonstrates superior performance without any hyperparameter tuning or pretraining. The employed parameters are summarized in Supplementary Table S8. LEFTNet is chosen as the main backbone due to its direct comparability with React-OT. React-OT, however, employs an object-aware conditioning on the reactant and product structure, which substantially increases both computational and memory demands. The main reason is the construction of a fully connected graph over all fragments, reactant, product, and TS. Thus, a seemingly small reaction with only 15 atoms per fragment results in a graph comprising 45 nodes. Since the number of edges in a fully connected graph scales quadratically with the number of nodes, this conditioning severely restricts the size and complexity of the backbone that can be used. In contrast, AEFM focuses solely on reconstructing the TS without requiring explicit reaction context, enabling the use of a more powerful and scalable backbone architecture. This difference is also reflected in the training time. AEFM requires approximately 18 seconds per epoch, resulting in a total training time of about 3 hours for 600 epochs and 5 hours for 1000 epochs. In comparison, OAReactDiff and React-OT require roughly 2 minutes

per epoch, and with a total of 2200 epochs, their full training time reaches approximately 73 hours. All performance measurements were obtained using an NVIDIA A40 GPU.

Table S6: Statistical performance of AEFM on React-OT.

Approach	RMSD (Å)		$\lvert\Delta E_{\mathrm{TS}}\rvert$ (kcal mol^{-1})	
	Mean	Median	Mean	Median
React-OT + AEFM (seed=8)	0.188	0.089	3.390	0.745
React-OT + AEFM (seed=13)	0.187	0.089	3.334	0.779
React-OT + AEFM (seed=42)	0.188	0.088	3.341	0.793

Table S7: Overview of hyperparameters.

(a) Model hyperparameters.

Parameter	Value
Message passing layers	6
Equivariant readout layers	1
Hidden features	196
Radial basis functions	96
Cutoff radius	10 Å
Learning rate	10^{-4}
Batch size	64
σ_{FM}	0.05
Optimizer	AdamW
Learning rate scheduling	No scheduler

(b) Training hyperparameters for low-fidelity sources.

Method	σ	Epochs
xTB CI-NEB	0.19	1000
React-OT (xTB)	0.12	600
React-OT	0.11	600

Illustrative cases of AEFM refinements

Supplementary Figure S2 illustrates two reactions with varying quality of initial React-OT TS predictions alongside their AEFM-refined counterparts. For the first reaction, the initial React-OT prediction is poor, exhibiting an RMSD of 0.46 Å, and AEFM refinement further increases the deviation to 0.83 Å. For the second reaction, React-OT produces an excellent initial guess (RMSD 0.04 Å), which is further improved by AEFM to 0.01 Å.

Table S8: Hyper-parameters of EquiformerV2 (76M).

Parameter	Value
Optimizer	AdamW
Learning rate scheduling	No scheduler
Learning rate	10^{-4}
Batch size	32
Weight decay	10^{-3}
Dropout rate	0.1
Stochastic depth	0.05
Model EMA decay	0.999
Gradient clipping norm threshold	100
Cutoff radius (Å)	5.0
Number of radial bases	512
Dimension of hidden scalar features d_{edge}	128
Maximum degree $L_{\max}$	6
Maximum order $M_{\max}$	2
Number of layers	6
Embedding dimension d_{embed}	128
$f_{ij}^{(L)}$ dimension $d_{\text{attn\_hidden}}$	128
Number of attention heads h	8
$f_{ij}^{(\alpha)}$ dimension $d_{\text{attn\_alpha}}$	32
Value dimension $d_{\text{attn\_value}}$	16
Hidden dimension in feed forward networks d_{ffn}	512

In depth analysis for GFN2-xTB samples

Supplementary Figure S3 illustrates the performance of AEFM on GFN2-xTB CI-NEB TS guesses. Notably, even samples with large initial RMSD and energy errors show substantial improvement (panel S3a). In contrast to the behavior on the React-OT samples, the mean energetic improvement appears to be dominated by outliers, with cases of small RMSD showing the least improvement and those with RMSD above 1.0 Å showing stronger gains (panel S3c). Upon visual inspection of these high RMSD samples, it becomes evident that many of the GFN2-xTB TS guesses are fragmented into multiple substructures, despite the corresponding reference TS being a single connected entity. In these cases, AEFM successfully reconstructs the molecular connectivity, yielding structurally realistic and chemically plausible geometries. Although the refined TS structures may still deviate from the target

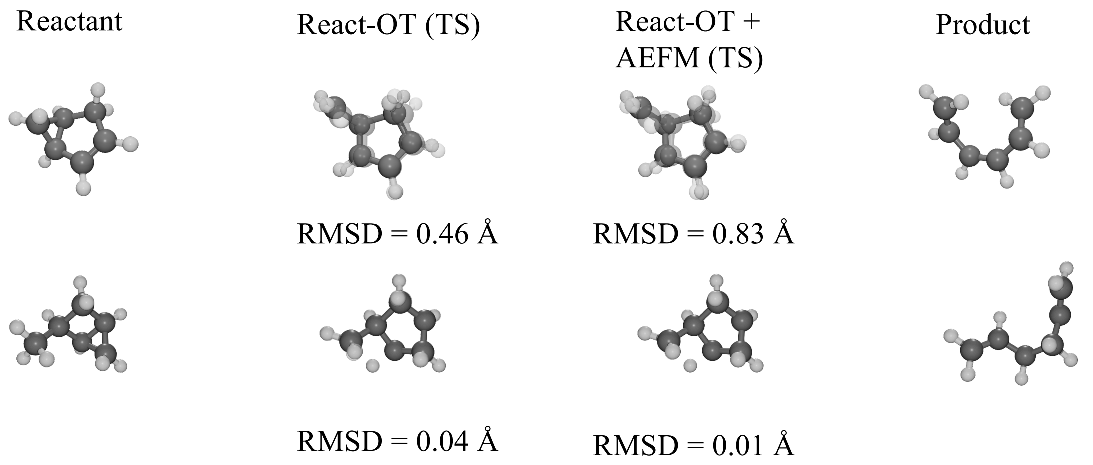

Figure S2: **Example reactions showing reactant, TS predictions from React-OT, the AEFM-refined TS, and the product.** The predicted TS is overlaid transparently on the reference TS from the Transition1x dataset. First reaction with a poor initial TS guess from React-OT, further worsened by AEFM. Second reaction with a good initial TS guess from React-OT, further improved by AEFM.

configuration, they exhibit a substantial energetic improvement, converging much closer to the reference energy. The fraction of improved samples follows the same trend as before and decreases with increasing RMSD, indicating that high-RMSD structures lie outside the training distribution. Consequently, while the model cannot fully reconstruct the intended TS in these cases, it can still correct structurally implausible geometries, thereby improving their physical realism. This effect is exemplified in Supplementary Figure S4, where the GFN2-xTB TS guess contains a detached hydrogen atom that AEFM correctly reattaches, resulting in a more faithful representation of the intended TS. This correction results in a substantial energetic improvement ($\Delta E \sim -80$ kcal mol^{-1}), although the refined structure still retains a relatively large RMSD of 1.39 Å. Such artifacts are not observed for the React-OT-generated structures, consequently its improvement trend is instead dominated by low-RMSD cases.

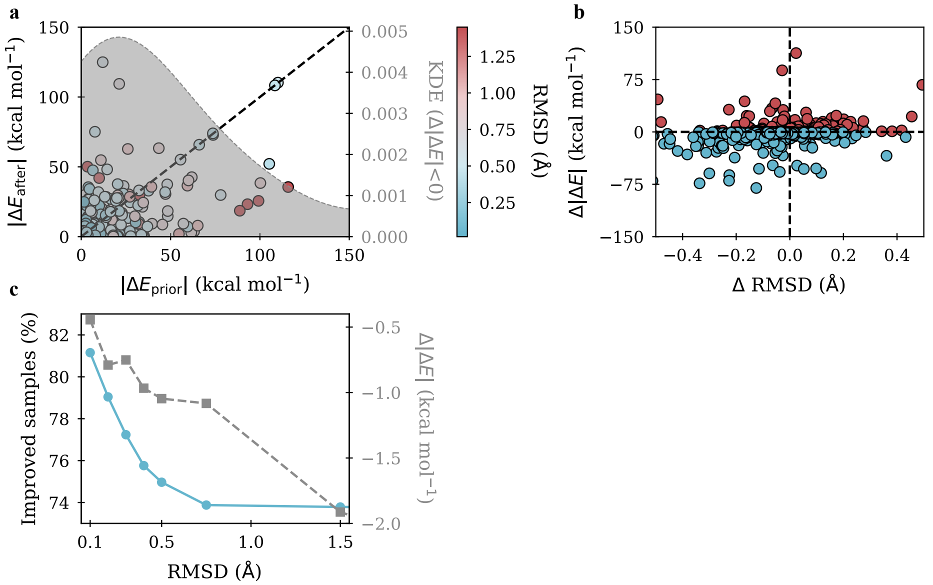

Figure S3: **Relationship between energetic and structural changes in AEFM refinements, with a focus on outliers and correlation trends.** **a** Energetic differences of AEFM-refined structures versus initial GFN2-xTB predictions on the left y-axis. Points below the diagonal line indicate improved agreement with the reference TS, while points above reflect increased deviation. On the right y-axis, the KDE of improvement weighted by the improvement magnitude is shown. **b** Energetic vs. geometric changes resulting from the application of AEFM. The bottom-left quadrant indicates improvements in both structural and energetic similarity, while the bottom-right quadrant reflects improved energy alignment accompanied by reduced structural similarity. Blue points indicate an energetic improvement, while red points correspond to increased dissimilarity. **c** Improvement rate (left y-axis in blue) and mean reduction in energy error (right y-axis in grey) as a function of the initial GFN2-xTB RMSD.

Quantum chemical validation

To verify that the DFT-optimized TSs correspond to the intended reactions, we evaluate two criteria: (i) the structural deviation from the P-RFO–converged TSs in Transition1x, and (ii) the reactant and product minima obtained from intrinsic reaction coordinate (IRC) calculations. Supporting Table S10 reports the first metric, showing that, in terms of median RMSD, all methods typically converge to the correct TS. For React-OT and React-OT + AEFM, 85% of the optimized TSs match the Transition1x reference, indicating that both approaches provide reliable starting structures for downstream DFT optimization. For GFN2-xTB, the addition of AEFM increases both the fraction of successfully converged structures,

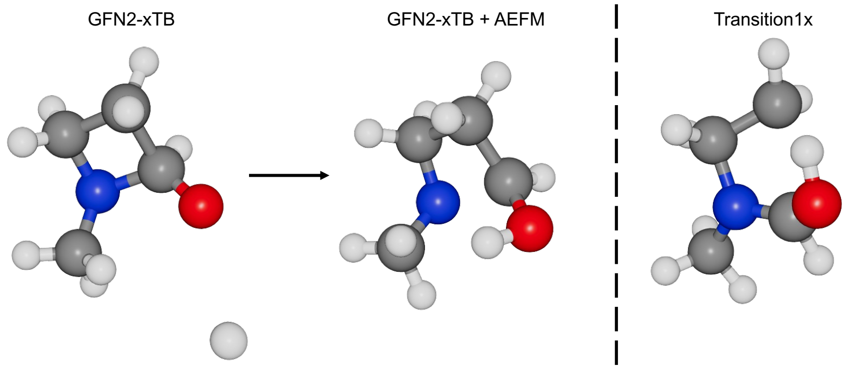

Figure S4: **Energetic outlier at the GFN2-xTB level.** Shown is a TS guess obtained from a CI-NEB calculation at the GFN2-xTB level of theory, alongside its AEFM-refined counterpart. For reference, the corresponding ground-truth TS structure from the Transition1x dataset is also displayed. The GFN2-xTB TS guess exhibits a disconnected hydrogen atom, which is accurately reattached by AEFM, yielding a more chemically realistic structure that closely resembles the reference TS. This correction results in a substantial energetic improvement ($\Delta E \sim -80$ kcal mol^{-1}), although the refined structure still retains a relatively large RMSD of 1.39 Å.

and the structural similarity to the reference by 6%. Table S11 summarizes the IRC results using the median RMSD between the minima obtained from IRC and those associated with the Transition1x TSs. Interestingly, the apparent success rate for Transition1x is lower, despite the TSs themselves showing high structural agreement (mean RMSD = 0.044 Å and 91% matching under a 0.05 Å threshold). The primary reason is that IRC often identifies alternate conformers while still following the correct reaction path. An example of this behavior is shown in Supporting Figure S5, where the same reaction is recovered but a different product conformer is found. React-OT and React-OT + AEFM exhibit IRC success rates comparable to those of the Transition1x reference, confirming the high quality of these ML-generated structures. Likewise, GFN2-xTB + AEFM achieves similarly strong performance, demonstrating that AEFM reliably enhances xTB-level initializations for downstream DFT tasks.

Table S9: Total CPU hours required for TS refinement using the P-RFO algorithm implemented in the Sella package with 48 CPU cores.

**Method**	**CPU Hours**
xTB CI-NEB	1430
xTB CI-NEB + AEFM	506
React-OT	455
React-OT + AEFM	439

Table S10: RMSD between DFT-converged TS obtained from model-generated TS structures and from Transition1x.

Approach	RMSD (Å)		RMSD[a] (Å)		Matched TS[b] (%)
	Mean	Median	Mean	Median	
React-OT	0.083	0.003	0.056	0.001	85
React-OT + AEFM	0.085	0.001	0.056	0.000	85
xTB CI-NEB	0.110	0.002	0.064	0.001	70
xTB CI-NEB + AEFM	0.148	0.001	0.105	0.000	76

[a] RMSD is only computed for heavy atoms.
[b] Considered same structure if RMSD below 0.05 Å.

Out-of-distribution bevahior

To further assess the performance of AEFM on out-of-distribution datasets, three small benchmark sets were considered. The first contains 18 distinct Diels–Alder reactions, where the initial TS structures were generated with the Connectivity Transition State (CTS) method combined with the universal force field (UFF) and subsequently optimized using the P-RFO algorithm at the PM6 level of theory. The test set contains previous unseen elements, like silicon, fluor, and sulfur, making it a challenging test for the model. The PM6 TSs were optimized using the P-RFO algorithm at the ωB97X/6-31G(d) level of theory to maintain consistency with the Transition1x dataset, with the UFF TSs serving as initial guesses. To evaluate AEFM, the mean RMSD of the TS guesses was computed, and a dedicated model was trained on samples from the Transition1x dataset with $\sigma = 0.31$. TS optimizations failed for three of the 18 reactions, so the statistics in Supplementary Table S12 are reported for the remaining 15 reactions. Interestingly, EquiformerV2 shows an

Table S11: Median RMSD between IRC and Transition1x minima.

Approach	$RMSD_{reactant}$ (Å)	$RMSD_{reactant}{}^{a}$ (Å)	$RMSD_{product}$ (Å)	$RMSD_{product}{}^{a}$ (Å)	Matched Reactions[b] (%)
Transition1x	0.049	0.024	0.087	0.027	59
React-OT	0.041	0.012	0.096	0.037	56
React-OT + AEFM	0.058	0.021	0.151	0.040	56
xTB CI-NEB	0.063	0.026	0.197	0.044	48
xTB CI-NEB + AEFM	0.055	0.024	0.157	0.035	56

[a] RMSD is only computed for heavy atoms.
[b] Considered same reaction if heavy atom RMSD for reactant, TS, and product are all below 0.1 Å.

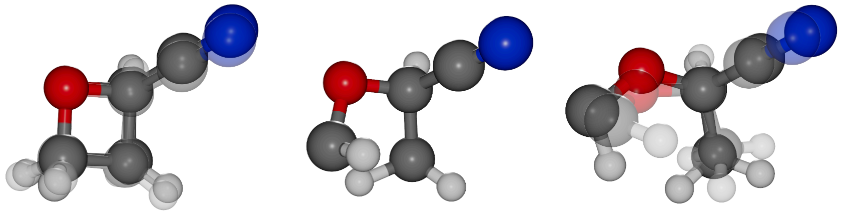

Figure S5: **Overlay of the reaction retrieved via P-RFO optimization followed by IRC and the original reaction from the dataset.** The IRC-derived structures are shown transparently. Although the TS coincides nearly perfectly with the reference (RMSD = 0.0004 Å), the reactant and product deviate more substantially (RMSD = 0.143 Å and 0.847 Å, respectively).

increase in the mean energetic deviation when evaluated on the full OOD benchmark. To investigate this behavior, we separated the benchmark into two subsets, one containing only HCNO chemistry and another including samples with out-of-distribution elements such as Si, S, and F. Across both subsets, the trend becomes clear, while both models perform well on the HCNO subset, performance degrades substantially once unseen chemical elements are introduced. This outcome is expected, as the models were never trained on these atom types and therefore lack the learned representation needed to handle them.

As a second test, 500 TS guesses curated by KinBot and distributed with the Sella package were examined, all of which correspond to HCO reaction chemistry. In this case, only the initial guesses were available, and the optimized TS structures were obtained by us through P-RFO optimization at the ωB97x/6-31G(d) level of theory. Out of the 500 initial samples, 473 converged to valid TSs characterized by a single negative frequency. Based on

the mean RMSD between the initial guesses and the optimized structures, an AEFM model was trained on Transition1x with $\sigma = 0.28$. Analysis of the chemical sum formulas revealed that 284 of the 473 cases represent entirely new chemistry, while the remaining 189 share a formula with Transition1x reactions but none had an RMSD below 1 Å to any Transition1x TS.

To extend the evaluation into a more chemically complex domain, we applied AEFM to a ruthenium-catalyzed ethylene hydrogenation dataset (CaTS). The dataset contains 402 CI-NEB TSs computed using the same workflow as Transition1x, with the B3LYP/def2-SVP level of theory chosen to appropriately describe the transition metal. The dataset was split into 295 training, 32 validation, and 75 test reactions. GFN2-xTB was used to generate initial TS guesses for the test set, yielding 66 converged TS guesses. Using the mean RMSD between initial guesses and optimized geometries, an AEFM model pretrained on Transition1x was finetuned on the 295 Ru-catalysis structures with a noise level of $\sigma = 0.17$. Single-point energies for the refined AEFM geometries were computed at the B3LYP/def2-SVP level. Supplementary Table S12 summarizes the performance of AEFM on all three out-of-distribution test sets.

Metrics

The RMSD for molecules is determined by first aligning the molecules $\mathbf{x}_1$ and $\mathbf{x}_2$ using the Kabsch algorithm and then computing:

$$\begin{aligned} \mathrm{RMSD}(\mathbf{x}_1, \mathbf{x}_2) &= \sqrt{\frac{\sum_{i=1}^{N} \|\mathbf{x}_{1,i} - \mathbf{x}_{2,i}\|^2}{N}} \\ &= \sqrt{\frac{\sum_{i=1}^{N} \sum_{j\in\{x,y,z\}} (x_{1,i,j} - x_{2,i,j})^2}{N}} \end{aligned} \tag{S1}$$

with N denoting the number of atoms. Note that this definition differs from the one used in React-OT, where the RMSD is normalized by $3N$ instead. To access the difference in barrier

Table S12: Out-of-distribution performance of AEFM.

Approach	RMSD (Å)		$\lvert\Delta E_{\mathrm{TS}}\rvert$ (kcal mol^{-1})	
	Mean	Median	Mean	Median
CTS UFF	0.429	0.404	51.024	41.503
CTS UFF + AEFM	0.414 (↓3%)	0.213 (↓47%)	49.012 (↓4%)	21.687 (↓48%)
CTS UFF + AEFM [b]	0.385 (↓10%)	0.226 (↓44%)	55.959 (↑10%)	15.461 (↓63%)
CTS UFF[a]	0.428	0.412	48.192	39.717
CTS UFF + AEFM [a]	0.367 (↓14%)	0.202 (↓51%)	26.792 (↓44%)	17.776 (↓55%)
CTS UFF + AEFM [a,b]	0.353 (↓17%)	0.176 (↓57%)	21.208 (↓56%)	4.933 (↓88%)
KinBot	0.491	0.422	49.569	36.198
KinBot + AEFM	0.505 (↑3t%)	0.460 (↑9%)	22.670 (↓54%)	14.317 (↓60%)
KinBot + AEFM [b]	0.459 (↓7%)	0.394 (↓7%)	17.903 (↓64%)	8.894 (↓75%)
KinBot[c]	0.452	0.356	42.198	32.480
KinBot + AEFM [c]	0.488 (↑8%)	0.402 (↑13%)	25.705 (↓39%)	19.099 (↓41%)
KinBot + AEFM [b,c]	0.421 (↓7%)	0.328 (↓8%)	18.849 (↓55%)	9.302 (↓71%)
CaTS xTB	0.312	0.282	14.614	12.544
CaTS xTB + AEFM [b]	0.247 (↓21%)	0.207 (↓27%)	8.996 (↓38%)	7.822 (↓38%)

[a] Considering only 12 reactions with HCNO chemistry.
[b] Results obtained using an out-of-the-box EquiformerV2 model.
[c] Considering only 284 reactions with sum formulas not present in the Transition1x dataset.

height, the electronic energy V of each sample TS structure is computed and the MAE is defined as

$$\mathrm{MAE} = \frac{1}{M}\sum_{i}^{M} \lvert V(\mathbf{x}_i) - V(\hat{\mathbf{x}}_i)\rvert \tag{S2}$$

with $\hat{\mathbf{x}}_i$ as the predicted TS and $\mathbf{x}_i$ as the corresponding database TS and M as the total number of samples. To compare the distribution of bond lengths in the predicted structures with those in the reference data, we use the Wasserstein-1 distance. Given two one-dimensional empirical distributions p and q over bond lengths with cumulative distribution functions P and Q, respectively, the Wasserstein-1 distance is defined as:

$$W_1(p,q) = \int_{-\infty}^{\infty} \lvert P(x) - Q(x)\rvert \, dx. \tag{S3}$$

The Wasserstein-1 distance is computed separately for each bond type in the dataset and subsequently averaged across all types. As a metric that quantifies the minimal effort re-

quired to transform one distribution into another, it is particularly well-suited for capturing differences in geometric structure distributions, such as bond lengths.
